# Choroid plexus failure in the Kearns-Sayre syndrome

**DOI:** 10.1186/1743-8454-7-14

**Published:** 2010-08-23

**Authors:** Reynold Spector, Conrad E Johanson

**Affiliations:** 1 Department of Medicine, Robert Wood Johnson Medical School, Piscataway, NJ, 08854 USA; 2 Department of Neurosurgery, Alpert Medical School at Brown University, Providence, RI, 02903 USA

## Abstract

The Kearns-Sayre syndrome is a mitochondrial disorder (generally due to mitochondrial DNA deletions) that causes ophthalmoplegia, retinopathy, ataxia and brain abnormalities such as leukoencephalopathy. In this syndrome, the choroid plexus epithelial cells, unlike brain cells, are greatly enlarged and granular, consistent with their inability to adequately transport folate from blood into cerebrospinal fluid (CSF), and homovanillic acid (a dopamine metabolite) from CSF into blood. This inability to transport folates from blood into CSF (and brain) adequately, causes cerebral folate deficiency that can be partially reversed by very high doses of reduced folates. The Kearns-Sayre syndrome is a disease that interferes with key choroid plexus functions and is a cause of generalized choroid plexus failure.

## Introduction

Besides secreting most of the cerebrospinal fluid (CSF), the choroid plexus (CP) epithelial cells, a locus of the blood-CSF barrier, like renal tubule cells, have myriad other functions. These include the active transport of essential micronutrients into CSF from blood, e.g., methyltetrahydrofolic acid (MeTHF) and ascorbic acid (AA) [1]. From CSF, these micronutrients enter mammalian brain [1]. The CP also transports many exogenous chemicals and waste products of metabolism, e.g., homovanillic acid (HVA) an end product of dopamine metabolism in brain, out of CSF.

In recent years, the mechanisms responsible for some of these functions of the CP have been worked out on a molecular basis. For example, the vectorial transport of MeTHF (the principal folate in plasma and CSF) from blood into CSF via the CP is due to a three-part active transport system in series: first, the folate receptor alpha (FRα) transfers MeTHF from blood into CP endosomes; then MeTHF is released from the endosomes into the CP cytoplasm by the proton-coupled folate transporter (PCFT); and finally MeTHF is released from the cytoplasm into CSF by facilitated diffusion via the reduced folate carrier (RFC) [1]. Another example is AA that is transported into CP from blood by the active sodium-dependent vitamin C-2 transporter (SVCT-2); how AA effluxes from CP into CSF is unknown. It is worth noting that the concentrations of vitamin C and MeTHF in CSF are about 3-4 times higher than the plasma concentrations, thus highlighting the active (energy-requiring) nature of these processes [1]. Finally, HVA is transported by CP out of CSF by the organic acid transporter 3 (OAT-3).

The principal objective of this paper is to answer a question we asked about thirty years ago: Can the CP fail as an organ in a generalized way in disease states [2]? If so, we predicted then that the clinical result would be devastating, since the CP has essential functions [2]. We think that the Kearns-Sayre syndrome (KSS) is apparently the first clear example of generalized CP failure.

## Discussion

In humans, there are now examples of specific CP functions that fail as a result of inherited and acquired conditions. For example, there are two different genetic causes of inability to pump MeTHF from blood into CSF via CP. These can be termed human knockouts (KOs). This can be due to KO of either FRα or PCFT [1,3]. These human KOs, as expected, have cerebral folate deficiency (CFD) and devastating neurological syndromes. An acquired cause of CFD is the formation of antibodies to the FRα [1]. On the other hand, KO of SVCT-2 (that transports AA into CSF) in animals and humans is not compatible with life [1].

The above examples are extremely revealing but do not answer the question about generalized CP failure - akin to renal failure - secondary to diffuse damage to the CP epithelial cells [2]. However, in recent years, a specific example of CP failure has been described in KSS [4-9]. KSS is caused by various abnormalities of the mitochondrial genome, often due to deletions of ~5 kb [4-9]. KSS often manifests itself with progressive external ophthalmoplegia, pigmented retinopathy, heart block, elevated CSF protein and cerebellar ataxia. However, the deletions or other DNA abnormalities do not interfere with the abnormal mitochondrial DNA's ability to replicate [4-8]. In humans, the normal mitochondrial genome is a circular double-stranded DNA helix of 16.5 kb that encodes 13 polypeptides and 22 t-RNAs and 2 ribosomal RNAs [8]. There are 2-10 copies of circular DNA per mitochondrion, an organelle that turns over ~ every 30 days. In the KSS syndrome during very early development, a deletion (or other abnormality) occurs in the mitochondrial DNA that is propagated in various tissues (heteroplasmy) like CP and muscle [8]. Heteroplasmy is defined as the presence of both normal and abnormal circular DNA double helices in mitochondria in a cell. When the heteroplasmy reaches ~30% or more abnormal DNA circles in the mitochondria of a cell, there are changes in that cell's structure and function due to the abnormal mitochondrial DNA [4-8]. As might be expected, sufficient heteroplasmy would lead to insufficient ATP production, a principal function of mitochondria.

In KSS, there are prominent anatomical changes in human CP epithelial cells that are greatly enlarged and full of eosinophilic granules [4]. *In vivo*, in humans with KSS one known functional consequence is the inability to transport adequate amounts of MeTHF into CSF with consequent CFD [4-7,9]. Most patients have extremely low MeTHF CSF concentrations even in the face of a normal plasma concentration [4-7,9]. In one series of patients (N = 6), the average CSF MeTHF concentration was 8 nM (normal range 50-134 nM) with an average plasma folate concentration of 17 nM (normal range 10-30 nM) [9].

Unlike MeTHF, the HVA concentrations in CSF are elevated in KSS (by about a factor of 2 above the upper limit of normal, N = 6) almost certainly due to decreased clearance of HVA from CSF, presumably by the CP epithelial OAT-3 [6,9]. There is no evidence for increased dopamine turnover in brain that would also explain the increased CSF HVA concentration [9]. One hypothesis for these abnormalities in KSS is inadequate provision of energy substrate (ATP) that drives the CSF-inward transport of MeTHF and the CSF-outward transport of HVA [10,11]. However, other potential explanations are possible. What is certain, though, is that an inward CP transport system (MeTHF) and an outward clearance system (HVA) are abnormal in KSS. Another abnormality in KSS is an elevated CSF protein. We posit that the increased protein concentration could result from leaky CP tight junctions or reduced CSF turnover rate (bulk flow) that results from curtailed ATP-driven CSF formation [10].

Associated with the low CSF MeTHF and the raised HVA and CSF protein in KSS, is a devastating progressive neurological disorder that begins before the age of twenty. KSS manifests itself clinically with ophthalmoplegia, retinopathy, ataxia and other abnormalities [4-7]. We postulate that the disease (KSS) begins to manifest itself when the heteroplasmy (abnormal mitochondrial DNA) reaches a threshold value [11]. In several cases, treatment with very high doses of reduced folate, with normalization of CSF MeTHF, has been beneficial - presumably ameliorating the CFD [4-7]. However, MeTHF treatment only partially reverses the signs and symptoms of KSS [4-7].

Thus, KSS can be considered a cause of diffuse CP failure. Presumably, beside MeTHF and HVA transport, many other important functions are abnormal. For example, we would predict AA concentrations in CSF will be low. Hence, if in fact the concentration of AA is low in CSF, KSS patients might benefit from high doses of intravenous dehydroascorbic acid (DHA). DHA enters brain via the glucose transporter at the blood- brain barrier (via the transporter glut 1) and is converted to AA in situ in brain [1].

## Conclusion

KSS is an example of a systemic mitochondrial disorder that affects the CP anatomically and physiologically. It is clear that the CP, like several other tissues (e.g., muscle) in KSS, suffers from excess mitochondrial DNA heteroplasmy. This presumably leads to insufficient ATP concentrations to drive cellular processes (e.g., active transport) in CP with consequent low CSF and CNS MeTHF concentrations, as well as elevated CSF HVA, and other so-far unmeasured CSF abnormalities. Our increased understanding of KSS has led to amelioration of the syndrome (with high doses of reduced folates), but there is clearly more work to be done. KSS is an example of the importance of basic research in unraveling complex clinical disorders.

## Abbreviations

AA: ascorbic acid (vitamin C); ATP: adenosine triphosphate; CFD: cerebral folate deficiency; CP: choroid plexus; CSF: cerebrospinal fluid; DHA: dehydroascorbic acid; FRα: Folate receptor alpha; HVA: homovanillic acid; KSS: Kearns-Sayre Syndrome; MeTHF: methyltetrahydrofolic acid; OAT-3: organic acid transporter 3

## Competing interests

The authors declare that they have no competing interests.

## Authors' contributions

RS and CEJ both analyzed the literature, wrote the manuscript and worked on the revision. Both authors have read and approved the final version of the manuscript.
